# Botulinum toxin in the treatment of sialorrhea in severe neurological patients with tracheotomy

**DOI:** 10.1002/brb3.3164

**Published:** 2023-07-17

**Authors:** Mengmeng Shao, Keyang Chen, Xiaoyun Wu, Jingjing Lin, Mingxia Jiang, Feinan Zhuo, Zhaojian Ying, Yuanyuan Huang

**Affiliations:** ^1^ Department of Rehabilitation The First Affiliated Hospital of Wenzhou Medical University Wenzhou China; ^2^ Department of Neurology The Second Affiliated Hospital and Yuying Children's Hospital of Wenzhou Medical University Wenzhou China; ^3^ Department of Rehabilitation The First Affiliated Hospital of Wenzhou Medical University Wenzhou China; ^4^ Department of Emergency The First Affiliated Hospital of Wenzhou Medical University Wenzhou China

**Keywords:** botulinum toxin type A (BTA), drooling, severe neurological patient, tracheotomy, ultrasound guidance

## Abstract

**Objective:**

To observe the clinical effect of botulinum toxin type A (BTA) injection into the salivary glands of the severe neurological patients with tracheotomy

**Methods:**

Seven patients with severe neurological disorders after tracheotomy and obvious drooling symptoms were enrolled. BTA was injected into bilateral parotid glands and submandibular glands under the guidance of ultrasound. Unstimulated salivary flow rate (uSFR) and Drooling Severity and Frequency Scale (DSFS) were used to evaluate drooling before injection, 1 week, and 4 weeks after injection. We compared the extubation time, time of changing from balloon cannula to metal cannula, hospitalization time and incidence of recurrent pulmonary infection between these patients and other patients accepted conventional curation.

**Results:**

(1) The drooling severity scale (DSFS‐S), the drooling frequency scale (DSFS‐F), the drooling frequency and severity scale total score (DSFS‐T) were significantly lower at 4 weeks after BTA injection compared to prior‐treatment (*p* < .001). (2) uSFR of 1 week and 4 weeks were both statistically decreased than the untreated condition (*p* < .001). (3) Compared with the conventional group, the time of changing from balloon cannula to metal cannula was shortened obviously (*p* < .05) and incidence of recurrent pulmonary infection was clearly decreased (*p* < .05) after BTA treatment

**Conclusion:**

Ultrasound‐guided BTA injection into salivary glands can effectively reduce saliva secretion. We also found that the time of changing cannula was shortened obviously and the incidence of recurrent pneumonia infection was reduced. BTA injection of salivary glands to cure drooling could advance to the clinical therapy in severe neurological patients after tracheotomy.

## Graphical abstract

Ultrasound‐guided BTA injection into salivary glands can effectively reduce saliva secretion. BTA injection of salivary glands to cure drooling could advance to the clinical therapy in severe neurological patients after tracheotomy.

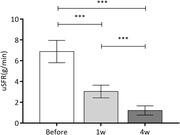

## 1 INTRODUCTION

Sialorrhea, also known as salivation or drooling, is the unintentional leakage of saliva from mouth or frequent swallowing discomfort caused by increased salivary gland secretion or dysphagia. It commonly occurs in patients with following diseases: neurological disorders including cerebral palsy (Hung et al., 2021), Parkinson disease (PD) (Arboleda‐Montealegre et al., 2021), traumatic brain injury (TBI) (Ko et al., 2013), stroke, and various degenerative brain (Anandan & Jankovic, 2021). Drooling leads to functional, social, physical problems, such as aspiration pneumonia (Banfi et al., 2015), dysphagia (Ou et al., 2015), dehydration, unpleasant odor (Suskind & Tilton, 2002), and emotional consequences. In addition, excessive salivation certainly increases the burden on caregivers. Traditional treatments for hypersalivation include oral anticholinergic medications, irradiation therapy and surgical intervention (Garcia Ron et al., 2012; Jeung et al., 2012). However, these treatments are poorly tolerated, often ineffective in a number of patients and are invasive accompanied with unacceptable side effects (Restivo et al., 2018). Recently, the percutaneous injection of BTA into salivary glands has been shown to be effective in abolishing sialorrhea in several neurological disorders (Restivo et al., 2018). In recent years, it was reported that the clinical application of botulinum toxin in the treatment of patients with salivation caused by brain injury, Parkinson's disease, cerebral infarction, etc was effective. (Ko et al., 2013; Ruiz‐Roca et al., 2019). The present pilot study evaluated the efficacy of botulinum toxin type A (BTA) injection into the parotid and submandibular glands under ultrasonic guidance in patients with severe drooling with neurological diseases after tracheotomy.

## 2 MATERIALS AND METHODS

### 2.1 Patients

Twenty‐one patients with tracheotomy and severe neurological disorders of different etiologies suffered from drooling from June 2020 to December 2020 were selected for sialorrhea. Out of these 21 patients with sialorrhea, 7 subjects (3 men and 4 women; mean age: 64.43 ± 14.14 years) who had experienced high frequency and severity of hypersalivation and drooling in the preceding 6 months and satisfied all the inclusion/exclusion criteria were enrolled in the study. The diagnoses were cerebral hemorrhage in two, multiple cerebral infarction in one and medullary infarction in four (Table 1).

**TABLE 1:** Characteristics of subjects (*n* = 7).

Characteristics	Value
Gender (male:female)	3:4
Age (year)	64.43 ± 14.14
Diagnosis	
cerebral hemorrhage	2
multiple cerebral infarction	1
medullary infarction	4

### 2.2 Inclusion criteria

① The diagnosis criteria according to the Fourth National Cerebrovascular Conference, confirmed by head CT or MR, and severe neurological patients with tracheotomy; ② disease course within 3 months; ③ accompanied by salivation; ④ severity of drooling ≥ grade 3; ⑤ agreed with salivation assessment; ⑥ accept BTA injection and agree to participate in the study.

### 2.3 Exclusion criteria

① Allergic to botulinum toxin; ② with serious liver, kidney, blood diseases and other primary diseases; ③ with tumor; ④ cricopharyngeal muscle spasm; ⑤ cognitive disability;⑥ take medicines that treat salivation in recent month or take any medicines that might cause salivation.

**FIGURE 1:**
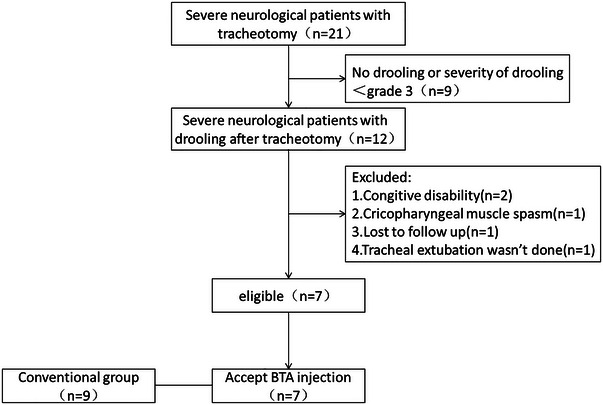
Flowchart of study patients.

As shown in Figure 1, a total of 21 patients were screened and 7 eligible patients were eventually recruited. However, 9 patients’ drooling severity was not over than grade 3 and did not reach the inclusion criteria, 2 patients were excluded because of cognitive disability, 1 patient was cricopharygeal muscle spasm and thus did not complete BTA injection and 1 patient's tracheal extubation was not done. We could not follow up one patient and he was excluded.

### 2.4 Treatment

The patients were routinely treated with anti‐infection, sputum resolution and asthma relief, nutritional support, and other conventional medications according to their condition after admission. At the same time, they accepted functional dysphagia therapy, respiratory function training, expectoration treatment, and speaking valve training. The conventional group and BTA group patients both accepted the above therapy.

Ultrasound‐guided BTA injection therapy: For each patient, 100 units of botulinum toxin type A (BTA) were diluted with 2 mL of normal saline so that 0.5 mL was equated to approximately 25 units and 1 mL was equated to 50 units. Then a qualified senior doctor used a 38 mm oral injection needle to perform the ultrasound‐guided BTA injection. A total of 1.2 mL (60 units BTA) was injected into each parotid gland at four sites and 0.8 mL (40 units BTA) into each submandibular gland at two sites, totaling 2 mL (100 units BTA) per patient.

### 2.5 Assessment

#### 2.5.1 Drooling severity and frequency scales (DSFS)

Investigators used the Drooling Severity and Frequency Scales (DSFS) to rate the change in drooling severity (using a 5‐point Likert scale from 1 to 5) and frequency (using a 4‐point Likert scale from 1 to 4), and the DSFS total score (maximum score of 9 and minimum score of 2 from the sum of the two subscales) (Jost et al., 2019; Thomas‐Stonell & Greenberg, 1988) before injection and 1 week and 4 weeks after injection. The detailed scale listed in Table 2.

**TABLE 2:** Drooling Severity and Frequency Scales (DSFS).

**Drooling Severity Scale (DSFS‐S)**	
1—Dry	Never drools
2—Mild	Only lips wet
3—Moederate	Wet on lips and chin
4—Severe	Drool extends to clothes wet
5—Profuse	Clothing, hands, tray, and objects wet
**Drooling Frequency Scale (DSFS‐F)**	
1—Never	
2—Occasionally	Not every day
3—Frequently	Part of every day
4—Constantly	

### 2.6 Unstimulated saliva flow rate (uSFR)

In the unstimulated state, the saliva was measured by weighing swabs. Before the measurement, the saliva in the oral cavity was cleaned with a medical swab. Dry medical swabs were weighed on an electronic scale and then they were placed on both sides of the patient's buccal mucosa and under the tongue, meanwhile avoiding chewing or swallowing. After 5 min, the wet medical swabs were taken out to get the weight. The weight increase of the swabs was used to calculate the salivary flow rate in g/min (the weight precision was 0.001 g) (Jost et al., 2019).

### 2.7 Extubation time, hospitalization time and other datas about the study

We observed the extubation time, hospitalization time, time of changing from balloon cannula to metal cannula, incidence of recurrent pulmonary infection in patients with severe neurological disease after tracheostomy who were not injected with BTA in the same period of last year. We also recorded above items of BTA group patients. We might find the differences between the two groups.

### 2.8 Statistical analysis

In this study, SPSS 23.0 statistical software was used for statistical analysis. Descriptive statistics were used to summarize baseline characteristics. Continuous variables were expressed as median (interquartile) and mean (standard deviation). Categorical variables were evaluated by calculating frequencies or percentages. Univariate analyses were conducted to analyze differences between two groups comparison were analyzed by Mann–Whitney *U* test, *t*‐test (continuous variable), and chi‐square test or the Fisher's exact test (categorical variable). *p* < .05 indicated that the difference was statistically significant.

## 3 RESULTS

### 3.1 Comparison of DSFS‐S, DSFS‐F, and DSFS‐T scores before and after BTA injection

**FIGURE 2:**
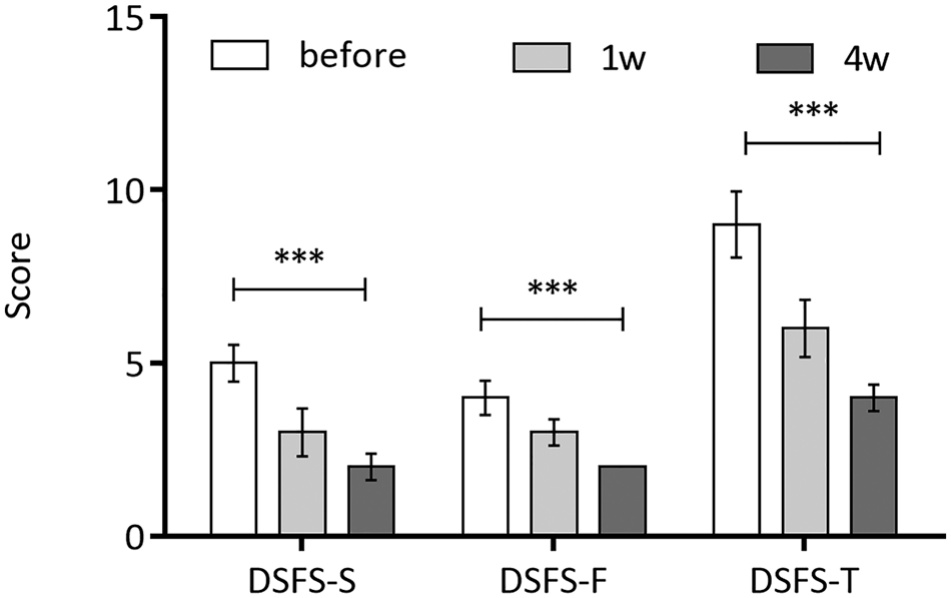
The Drooling Severity and Frequency Scales before BTA injection and at 1 week and 4 weeks after the injection.

The drooling severity scale (DSFS‐S), drooling frequency scale (DSFS‐F), and drooling severity and frequency scale total score (DSFS‐T) of the patients after 4w of BTA injection were significantly lower than those before injection (*p* < .01) (Figure 2).

The Drooling Severity and Frequency Scales and total score decreased significantly at 4 weeks after the injection (***p < .001).

### 3.2 Comparison of unstimulated salivary flow rate (uSFR) before and after BTA injection

**FIGURE 3:**
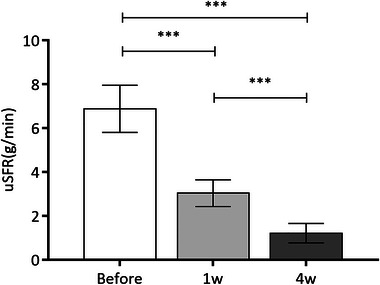
The unstimulated salivary flow rate (uSFR) before BTA injection and at 1 week and 4 weeks after the injection.

The uSFR of before injection and at 1 week and 4 weeks after injection were 6.880 ± 1.073 g/min, 3.037 ± 0.607 g/min, and 1.216 ± 0.441 g/min, respectively. The average unstimulated salivary flow rate of selected patients at 1 week and 4 weeks after BTA injection treatment was significantly lower than that before injection (*p* < .001); the average unstimulated salivary flow rate of patients at 4 weeks after injection was also significantly lower than that at 1 week after injection (*p* < .001). The difference was statistically significant (Figure 3).

After the injection, the uSFR at 1 week and 4 weeks decreased significantly (***p < .001). The uSFR at 4 weeks after injection reduced significantly compared to 1 week after injection (***p < .001).

### 3.3 Comparison of extubation time, hospitalization time, time of changing from balloon cannula to metal cannula (nonballoon canula), incidence of recurrent pulmonary infection in severe neurological patients after tracheotomy admitted to our department without BTA injection in the same period last year

The extubation time and hospitalization time of the BTA group were shortened than the conventional group (*p* > .05). The incidence of recurrent pulmonary infection was also obviously lower than those in the conventional group (*p* < .01) (Table 3). The most important item in our study we could find that the time of changing from balloon cannula to metal cannula in BTA group was distinctly lower than the conventional group (*p* < .05).

**TABLE 3:** The difference between BTA group and conventional group.

Characteristic	BTA group (*n* = 7)	conventional group (*n* = 9)	*p*
Gender (male:female)	3:4	7:2	.302
Age (year)	64.43 ± 14.14	79.44 ± 13.56	.387
Diagnosis			
Medullary infarction	4	1	
multiple cerebral infarction	1	3	
cerebral hemorrhage	2	2	
Alzheimer's disease	0	1	
Cerebral contusion and laceration	0	1	
Spinal cord injury	0	1	
Extubation time (day)	60.8 ± 16.0	142.0 ± 85.6	.091
Time of changing from balloon cannula to metal cannula (day)	34.7 ± 25.9	101.9 ± 77.6	.046
Hospitalization time (day)	78.4 ± 28.02	134.14 ± 95.20	.371
Incidence of recurrent pulmonary infection (%)	2 (28%)	9 (100%)	.005

## 4 DISSCUSSION

Drooling is a problem frequently faced by patients with neurological disorders. Salivary secretion is regulated by the autonomic nervous system. All salivary glands are dominated by sympathetic nerve fibers as well as parasympathetic nerve endings (Jost et al., 2019). Parasympathetic nerves provide the main motivation for glandular secretion, while sympathetic nerves regulate the composition of saliva. Nerve endings in the parasympathetic postganglionic system secrete acetylcholine, and block these receptor sites to inhibit neurostimulation of the salivary glands (Garrett, 1987; Jongerius et al., 2001).Pharyngeal sensory deficit or central interruption of normal swallowing reflexes inducing inadequate oromotor control would be the cause of drooling in brain injury patients (Hussein et al., 1998).

In recent years, BTA injection into the salivary glands has become a safe, minimally invasive and clinically effective method for the treatment of hypersalivation. Botulinum toxin to treat the nerve gland junction was first proposed in 1822 by Justinus Kerner. He mentioned severe dry mouth symptoms in patients with botulism, and thus suggested that the botulinum toxin could be used to treat sialorrhea (Erbguth, 1998). Animal studies began in the late 1990s and confirmed that BTA injection into salivary glands was effective. Subsequent clinical trials involving patients with cerebral palsy, Parkinson's disease and amyotrophic lateral sclerosis reported successful results.

Ultrasound guidance can accurately assess the structure of the head and neck, which allows precise needle positioning and minimizes irritation to surrounding tissues and vascular structure (Jongerius et al., 2003) thus enhancing the efficacy and safety of injection and reducing the incidence of adverse reaction. In our study, 10 sites of salivary glands were injected. There were no obvious injection complications or adverse reactions in the 7 related patients. We could not find the standard for the recommended dose of botulinum toxin for the treatment of salivation by now. In the previous study (Jongerius et al., 2001) the injected salivary glands were bilateral parotid glands and submandibular glands and the therapeutic dose of BTA was 100 U. Our study proved that the effect of ultrasound‐guided BTA injection into salivary glands on drooling was reliable.

It was showed that the drooling severity and frequency scale and total sum of drooling severity and frequency scale at 4 weeks after treatment were all obviously lower than untreated state. The unstimulated salivary flow rate was significantly improved after 1 week and 4 weeks of BTA injection and the difference was statistically significant (*p* < .001). BTA injection takes effect within 1 week, and the salivation symptoms can be significantly improved in 4 weeks. The time of changing from balloon cannula to metal cannula was clearly shortened after BTA injection. Not surprisingly, the incidence of recurrent pneumonia was significantly decreased. We took the BTA injection before changing cannula, drooling of patient was improved so as that the incidence of aspiration was decreased, and we could find that the time of changing cannula was shortened.

In conclusion, ultrasound‐guided injection of BTA can effectively reduce saliva secretion and improve salivation symptoms in patients with severe neurological disease after tracheotomy. BTA injection accompanied with functional dysphagia therapy, respiratory function training, expectoration treatment and speaking valve training can shorten extubation time and hospitalization time and reduce the risk of pulmonary infection during tracheotomy. We could find that BTA injection in salivary glands of the severe neurological patients after tracheotomy accompanied by sialorrhea might improve the condition and shorten course of disease and complete tracheal extubation earlier. The deficiency of this study listed as following: (1) The number of enrolled patients is relatively insufficient. (2) The observation time for BTA curative effect is short and the comparison of different BTA injection doses is not carried out. (3) The heterogeneous etiology of sialorrhea in the recruited patients. The above problems will be further improved in the follow‐up study.

## 5 CONCLUSION

Ultrasound‐guided BTA injection into salivary glands can effectively relieve the symptom of drooling. The time of changing cannula was shortened obviously and the incidence of recurrent pneumonia infection was reduced in our study. BTA injection of salivary glands to cure drooling could advance to the clinical therapy in severe neurological patients after tracheotomy and might be an effective method in these people.

The manuscript complies with all instructions to authors.

## AUTHOR CONTRIBUTIONS

MS: study conception and design, acquisition, analysis, and interpretation of data; perform the BTA injection under ultrasound, drafting the article and revising it critically for important intellectual content. KC: acquisition, analysis, and interpretation of data; drafting the article and revising it critically for important intellectual content. XW: acquisition, analysis, and interpretation of data; drafting the article and revising it critically for important intellectual content. JL: acquisition, analysis, and interpretation of data; drafting the article and revising it critically for important intellectual content. MJ: study conception and design, acquisition of data, analysis, and interpretation of data; drafting the article and revising it critically for important intellectual content. FZ: study conception and design, acquisition of data, analysis, and interpretation of data; drafting the article and revising it critically for important intellectual content. ZY: study conception and design, acquisition of data, analysis, and interpretation of data; drafting the article and revising it critically for important intellectual content. YH: study conception and design, acquisition of data, analysis, and interpretation of data; drafting the article and revising it critically for important intellectual content. The final manuscript was approved by all authors.

We declared that this manuscript has not been published elsewhere and is not under consideration by another journal.

## FUNDING INFORMATION

No funding support in this study.

## CONFLICT OF INTEREST STATEMENT

No potential conflict of interest relevant to this article was reported.

### PEER REVIEW

The peer review history for this article is available at https://publons.com/publon/10.1002/brb3.3164.

## Data Availability

The data underlying this article is available in the article.
